# Response of Freshwater Biofilms to Antibiotic Florfenicol and Ofloxacin Stress: Role of Extracellular Polymeric Substances

**DOI:** 10.3390/ijerph16050715

**Published:** 2019-02-27

**Authors:** Chaoqian Wang, Deming Dong, Liwen Zhang, Ziwei Song, Xiuyi Hua, Zhiyong Guo

**Affiliations:** Key Laboratory of Water Resources and Environment, College of New Energy and Environment, Jilin University, Changchun 130012, China; wangcq16@mails.jlu.edu.cn (C.W.); dmdong@jlu.edu.cn (D.D.); zhangliwen@jlu.edu.cn (L.Z.); songzw18@mails.jlu.edu.cn (Z.S.); huaxy@jlu.edu.cn (X.H.)

**Keywords:** EPS, enzyme, chlorophyll, fluoroquinolone, fluorinated antibiotic

## Abstract

Antibiotic residues have been detected in aquatic environments worldwide. Biofilms are one of the most successful life forms, and as a result are ubiquitous in natural waters. However, the response mechanism of freshwater biofilms to the stress of various antibiotic residues is still unclear. Here, the stress of veterinary antibiotic florfenicol (FF) and fluoroquinolone antibiotic ofloxacin (OFL) on freshwater biofilms were investigated by determining the changes in the key physicochemical and biological properties of the biofilms. The results showed that the chlorophyll a content in biofilms firstly decreased to 46–71% and then recovered to original content under the stress of FF and OFL with high, mid, and low concentrations. Meanwhile, the activities of antioxidant enzymes, including superoxide dismutase and catalase, increased between 1.3–6.7 times their initial values. FF was more toxic to the biofilms than OFL. The distribution coefficients of FF and OFL binding in extracellular polymeric substances (EPS)-free biofilms were 3.2 and 6.5 times higher than those in intact biofilms, respectively. It indicated that EPS could inhibit the FF and OFL accumulation in biofilm cells. The present study shows that the EPS matrix, as the house of freshwater biofilms, is the primary barrier that resists the stress from antibiotic residues.

## 1. Introduction

The ecological risk of antibiotics in the aquatic environment has attracted widespread concerns due to their wide production, use, and discharge. The abuse of antibiotics could lead to the rapid emergence of antibiotic-resistant bacteria and genes, and reduce their therapeutic potential against animal and human pathogens [1,2,3]. Florfenicol (FF) and ofloxacin (OFL) are chloramphenicol and fluoroquinolone antibiotics, respectively. Their consumption were 10,000 tons and 5110 tons in China in 2013, respectively [4]. Due to their relatively high consumption, discharge, and persistence, FF and OFL have been widely detected in various aquatic compartments [5,6]. Previous studies have demonstrated that the accumulation of FF and OFL residues in waters could cause direct toxicity to algae [7,8].

Natural biofilms are composed of a variety of autotrophic and heterotrophic microorganisms such as bacteria and algae cells, extracellular polymeric substances (EPS), and adsorbed organic and inorganic matter [9]. They are regarded as one of the most widely distributed and successful life forms on Earth [10]. Freshwater biofilms are typical natural biofilms that play fundamental roles in shaping the architecture of aquatic systems. They are sensitive to trace toxic substances and are commonly used to determine the effects of contaminants on various aquatic systems [11,12,13,14,15,16]. Biofilm EPS consist of a complex of polymers mainly produced by microorganisms and exhibit important functions during the formation and growth of microbial aggregates and the migration and transformation of substrates [17]. Proteins and polysaccharides are the primary components in EPS [18].

It has been found that antibiotics could affect the community compositions and activities of biofilm cells [19,20]. Chlorophyll a is well-known as an important property of algae vitality, and is intimately involved in all aspects of primary photosynthesis; its content can be used to estimate the primary productivity [20]. It was found that excessive cellular reactive oxygen species (ROS) produced by antibiotic stress could damage the microbial chloroplast membrane system and the chlorophyll a content of algae decreased under the stress of norfloxacin, amoxicillin, cefradine, streptomycin, and tetracycline [19,21,22,23,24,25]. Antibiotics could also induce oxidative stress and cause antioxidant responses for algae cell to adapt to the changes of the external environment. The antioxidant enzymes such as superoxide dismutase (SOD) and catalase (CAT) were described to be effective against ROS produced by oxidative stress [26]. It was reported that the activities of SOD and CAT enzymes increased in *Chlorella vulgaris* when exposed to streptomycin [19]. It was also found that the SOD and CAT activity in green algae substantially increased against the stress of sulfamethoxazole, erythromycin, clarithromycin, ciprofloxacin, cefradine, ceftazidime, and chlortetracycline [27,28,29]. Biofilms was considered to be less sensitive to antibiotics than their equivalent planktonic form due to EPS being able to reduce and even sequester antibiotics into the biofilm inside [30,31]. EPS content was found to increase under the stress of tetracycline, ciprofloxacin, and sulfadiazine [32,33]. The protection of EPS could help the microorganisms resist the environmental stress and maintain metabolic behavior [34]. It was reported that the EPS played an important role in alleviating the direct damage of the antibiotics sulfamethizole, tetracycline, and norfloxacin to the biofilm cells [30]. However, due to the wide varieties of antibiotics, studies on the stress of them on natural biofilms and the response of biofilms should be carried out more sufficiently in order to reveal the related mechanism.

The response of freshwater biofilms under the stress of fluorinated antibiotic FF and OFL was explored in this study. Specifically, it attempted to: (1) investigate the response of freshwater biofilms under FF and OFL stress by determining the changes in chlorophyll a content, antioxidant enzyme activities, and EPS content; (2) explore the protective role of EPS by carrying out the batch sorption of FF and OFL on EPS-free biofilms (biofilms without EPS) and intact biofilms (original); (3) reveal which substances dominated the interaction between EPS and antibiotics by measuring the three-dimensional excitation-emission matrix (3D-EEM) fluorescence spectroscopy and infrared spectroscopy (IR) of EPS.

## 2. Materials and Methods

### 2.1. Primary Reagents

Standards of FF and OFL were purchased from Dr Ehrenstorfer GmBH (Augsburg, Germany). The basic information on FF and OFL is listed in Table S1 in the Supplementary Materials. Spectrally pure KBr was purchased from Sigma-Aldrich, USA and oven-dried for 24 h at 150 °C before use. All of the other reagents and chemicals used were of analytical grade and purchased from Sinopharm Chemical Reagent Co., Ltd., Shanghai, China.

### 2.2. Biofilms Cultivation and Exposure Experiments

The lake water was collected from Nanhu Lake in Changchun, China. The information of the lake is given in Table S2. It was added to a group of 30-L glass tanks, and trace mineral salts were proportionally added referred to the previous study [35]. Biofilms were cultured with a set of glass slides under natural light at ambient temperature. According to the microscopic observations, the biofilms were dominated by *Chlorella* and *Characium* belonging to *Chlorophyta*, and were derived from collected lake water. After 30 days, two grams of the biofilms were scraped for characterization and sorption experiments. The lake water in the tanks was changed to Milli-Q water to reduce the disruption from the lake water on the results of exposure experiment. Antibiotic FF and OFL were separately added to these tanks. A stress group with a gradient of three antibiotic concentrations of 10 μg·L^−1^, 100 μg·L^−1^, and 1000 μg·L^−1^ and a control group without antibiotics were set. Biofilms were sampled at 0 h, 24 h, 48 h, 96 h, 144 h, and 192 h for determination of their chlorophyll a content, enzyme activities, and EPS properties.

### 2.3. Determination of Biofilm Chlorophyll a Content, Enzyme Activities, and EPS Properties

The determination of chlorophyll a content was done according to the previous study [36]. Two milliliters acetone and 20-mg dry weight biofilms were added to the glass slurry tube and thoroughly ground. The ground biofilms were transferred to the colorimetric tube to extract chlorophyll a with 10 mL of 90% acetone at 4 °C for 24 h. Then, the colorimetric tube was centrifuged at 3500× *g* for 15 min. The absorbance of supernatants was measured at 663 nm, 645 nm, and 630 nm using a UNICO UV-2100 spectrophotometer. The activities of CAT and SOD enzymes in the biofilms were measured under the stress of FF and OFL using the corresponding enzymatic colorimetric assay kits (Jiancheng Bioengineering Institute, Nanjing, China). ANOVA analysis was performed to compare the significant differences between the enzymatic activities in the stress and control groups.

Biofilm EPS was isolated using a cationic exchange resin (CER). The samples with the ratio of CER/biofilms (70 g/1 g) were stirred for 16 h at 600 revolutions per minute (rpm). Thereafter, CER was removed, and EPS-free biofilms were separated by centrifugation (4000× *g*, 20 min, 4 °C). The supernatants collected from centrifugation were purified with an 8000-Da semipermeable membrane to obtain EPS. The polysaccharide and protein content in EPS were measured by the phenol-sulfuric acid method [37] and the corresponding kits (Jiancheng Bioengineering Institute, Nanjing, China). 

Meanwhile, the EPS were freeze-dried for spectral analysis. The interactions between the EPS and the two antibiotics were analyzed using three-dimensional excitation-emission matrix (3D-EEM) fluorescence spectroscopy and an infrared spectrometer (IR, IRAffinity-1S, Shimadzu, Japan). The detailed procedures are provided in the Supplementary Materials.

### 2.4. Batch Sorption Experiment

The sorption of FF and OFL by intact biofilms and EPS-free biofilms was carried out. FF and OFL with initial concentrations of 0 to 2.5 mg·L^−1^ and 4 mg of intact biofilms or EPS-free biofilms were added to 40-mL vials. The vials were kept in the dark and shaken in a water-bath shaker at 150 rpm for 24 h at 25 °C. After that, the vials were centrifuged at 1000× *g* for 5 min, and the supernatants were passed through a 0.45-μm glass filter for the antibiotic determination.

### 2.5. Determination of Antibiotics

The FF content was determined by high performance liquid chromatography (LC-20AT, Shimadzu, Japan) equipped with a 5 μm × 4.6 mm × 250 mm Eclipse ODS-C18 column set at 36 °C and a UV detector at a wave number of 225 nm. The mobile phase was a mixture of water/acetonitrile (65:35, *v*/*v*) at a flow rate of 0.8 mL·min^−1^. The OFL content was determined by a fluorescence spectrophotometer (F-2700, Hitachi, Japan) using the emission spectrum. OFL fluorescence was measured at an excitation wavelength of 293 nm, emission wavelength of 220 to 550 nm, and voltage of 700 V. The slits for both excitation and emission were 5 nm, and the scan speed was 3000 nm·min^−1^ [38].

### 2.6. Data Analysis

The sorption amounts of FF and OFL onto intact and EPS-free biofilms were calculated by Equation (1):

*Q*_e_ = (*C*_0_ − *C*_e_) × *V*/*W* (1)

where *Q*_e_ is the sorption amount, mg·g^−1^; *C*_0_ is the initial concentration of antibiotics, mg·L^−1^; *C*_e_ is the concentration of antibiotics at equilibrium solution, mg·L^−1^; *V* is the volume of the sorption solution, L; and *W* is the dry weight of the biofilm samples, g.

Langmuir, Freundlich, and dual reactive domain (DRDM) models were employed to fit the sorption data. These models are expressed by Equations (2), (3), and (4):

Langmuir model:

*Q*_e_ = *Q*_max_*K*_L_*C*_e_/(1 + *K*_L_*C*_e_) (2)

Freundlich model:

*Q*_e_ = *K*_F_*C*_e_*^n^* (3)

DRDM:

*Q*_e_ = *K*_p_*C*_e+_*Q*_max_*K*_L_*C*_e_/(1 + *K*_L_*C*_e_) (4)

where *Q*_max_ is the Langmuir sorption maximum capacity, mg·g^−1^; *K*_L_ is the Langmuir equilibrium constant, L·g^−1^; *K*_F_ is the Freundlich equilibrium coefficient, (mg·g^−1^)·(mg·L^−1^)^−n^; *n* is the Freundlich nonlinear coefficient; and *K*_P_ is the partition coefficient of the linear component of DRDM, L·g^−1^.

Single-point distribution coefficients *K*_d_ were calculated at selected FF and OFL equilibrium concentrations to compare the sorption capacity of biofilm samples. *K*_d_ was expressed by Equation (5):

*K*_d_ = *Q*_e_/*C*_e_ (5)

The Stern–Volmer equation is used to quantify fluorescence quenching caused by FF and OFL. It is expressed by Equation (6):

*F*_0_/*F* = 1 + *K*_q_τ_0_[*Q*] = 1+*K*_Sv_[*Q*] (6)

where *F*_0_ is the initial fluorescence intensity; *F* is the fluorescence intensities at given FF or OFL concentrations; *K*_q_ is the quenching rate constant, L·mol^−1^·s^−1^; τ_0_ is the average lifetime (10^−8^ s) in the absence of the quenche; [*Q*] is the FF or OFL concentrations, mol·L^−1^; and *K*_Sv_ is the Stern–Volmer quenching constant, L·mol^−1^. Model fitting was conducted using Origin version 9.0 software (Origin Lab, Northampton, MA, USA). The analysis of variance was conducted using SPSS version 19.0 software (SPSS Inc., Chicago, IL, USA).

## 3. Results and Discussion

### 3.1. Changes in Biofilm Chlorophyll a Content, Enzyme Activities, and EPS Properties under FF and OFL Stress

**Figure 1:**
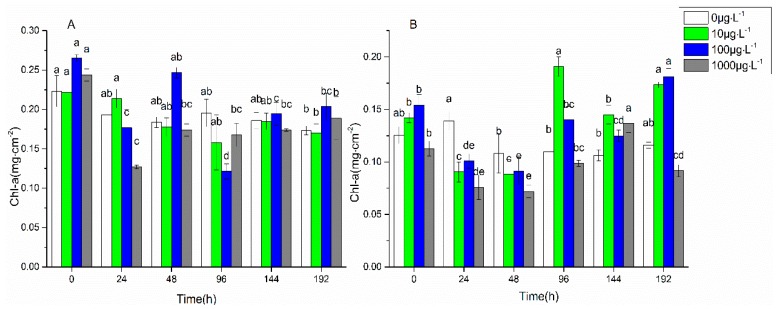
Changes of chlorophyll a content with time under florfenicol (**A**) and ofloxacin (**B**) stress. Marking the same letters, such as a and ab, represents no significant difference (*p* > 0.05), and marking different letters, such as a and b, represents a significant difference (*p* < 0.05).

The changes of chlorophyll a content with the stress time under 0 μg·L^−1^, 10 μg·L^−1^, 100 μg·L^−1^, and 1000 μg·L^−1^ of FF and OFL are shown in Figure 1. There was no significant change (*p* > 0.05) in the chlorophyll a content with the stress time in the control group. Under 10 μg·L^−1^ and 100 μg·L^−1^ of FF stress, the chlorophyll a content reached the lowest value at 96 h, which was 71% and 46% of the original content, respectively. Under 1000 μg·L^−1^ of FF stress, the chlorophyll a content reached the lowest value at 24 h, which was 52% of the original content. After the initial inhibition, the chlorophyll a content increased, and finally became close to the level of the control group (*p* > 0.05). Under 10 μg·L^−1^, 100 μg·L^−1^, and 1000 μg·L^−1^ of OFL stress, the chlorophyll a content decreased to the lowest values at 48 h, which were 62%, 59%, and 64% of the original content, respectively. However, there was no significant difference between the stress group and the control group (*p* > 0.05). The chlorophyll a content did not always decrease with the stress time. After the initial inhibition, the chlorophyll a content was found to recover to some extent.

The present results indicated that under the FF and OFL stress, the chlorophyll a in biofilms was inhibited in a short time. The possible reasons could be that FF and OFL promoted the production of ROS in algae cells, which directly destroyed the pigment structure [39]. In addition, ROS could induce chloroplast membrane damage, resulting in chlorophyll disorders in algal cells [21]. However, the results showed that the chlorophyll a content in biofilms could gradually recover under the antibiotic stress. It indicated that the biofilms have resistibility to the FF and OFL stress. It was found that *Chlorella vulgaris* showed a rapid recovery capability in the photosynthetic activity under FF stress [40,41]. It was also reported that biofilms showed some resistance under sulfamethizole, tetracycline, and norfloxacin stress [30]. The resilience was thought to be related to the antioxidant enzyme system [42].

**Figure 2:**
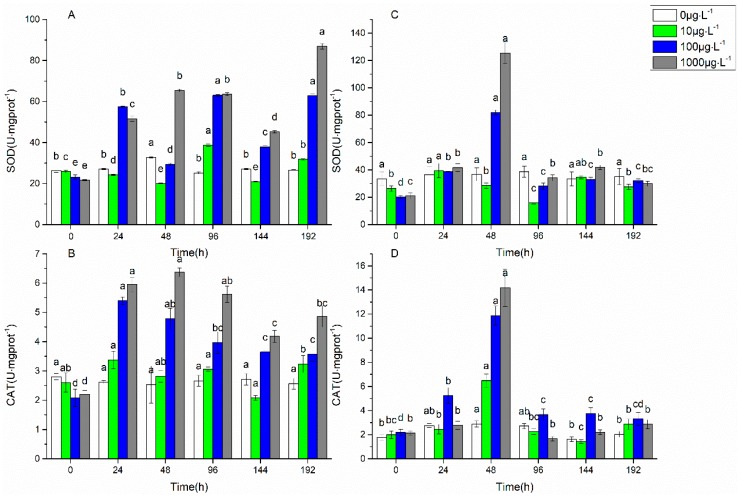
Changes of superoxide dismutase activity with time under florfenicol (**A**) and ofloxacin (**C**) stress; Changes of catalase activity with time under florfenicol (**B**) and ofloxacin (**D**) stress. Marking the same letters, such as a and ab, represents no significant difference (*p* > 0.05), and marking different letters, such as a and b, represents a significant difference (*p* < 0.05).

The antioxidant enzyme system is considered to be a shield for microorganisms to resist various external stresses. The changes of SOD and CAT activities with the stress time under 0 μg·L^−1^, 10 μg·L^−1^, 100 μg·L^−1^, and 1000 μg·L^−1^ of FF and OFL are shown in Figure 2. Under the stress of 10 μg·L^−1^, 100 μg·L^−1^, and 1000 μg·L^−1^ FF, the SOD activity increased from 24 h and reached the highest value at 96 h, 96 h, and 192 h, which were 1.5, 2.7, and 4.0 times of the original values, respectively, and the CAT activity reached the highest value at 24 h, 24 h, and 48 h, which were 1.3, 2.6, and 2.9 times of the original values, respectively. Under the stress of 10 μg·L^−1^, 100 μg·L^−1^, and 1000 μg·L^−1^ OFL, the SOD activity reached a maximum at 24 h, 24 h, and 48 h, which were 1.5, 4.1, and 6.0 times of the original values, respectively, and the CAT activity increased from 24 h and reached the highest value at 48 h, which were 3.3, 5.5 and 6.7 times of the original values, respectively. The decrease in SOD and CAT activities occurred at 96 h. The activities of SOD and CAT increased with the increase of FF and OFL concentrations.

The xenobiotic contaminants could induce oxidative stress and cause antioxidant responses for algae cells to adapt to the stress [43]. It was found that SOD and CAT activities in *Chlorella vulgaris* increased markedly under the stress of the antibiotic streptomycin, which could effectively remove ROS and reduce membrane damage [19]. It was also reported that SOD and CAT activities in *Chlorella pyrenoidosa* substantially increased to be against to the stress of ceftazidime and chlortetracycline [27,28]. In the present study, the activities of SOD and CAT reached the maximum at 48 h and decreased at 96 h under the OFL stress, but they still significantly higher than the initial enzyme activities (*p* < 0.05). It indicated that the antioxidant enzyme system of biofilms quickly responded to the OFL stress. Under the FF stress, the activities of SOD and CAT continued to maintain a high level after the increase, indicating that FF has a sustained strong stress on the biofilms. The previous study found that FF was more harmful to algae than OFL, due to FF as a protein inhibitor being more harmful to biofilm cells than OFL, which is as a nucleic acid inhibitor. In addition, the bioavailability of OFL is much lower than FF [8].

**Figure 3:**
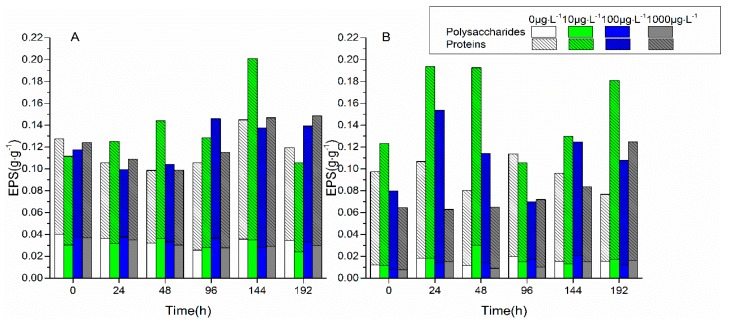
The content changes of extracellular polymeric substances with time under the stress of florfenicol (**A**) and ofloxacin (**B**).

The changes in EPS content with the stress time under 0 μg·L^−1^, 10 μg·L^−1^, 100 μg·L^−1^, and 1000 μg·L^−1^ of FF and OFL are shown in Figure 3. There was no significant change in EPS content compared with the stress and control groups. The EPS content increased rapidly under the stress of 10 μg·L^−1^ FF, and reached the highest value at 144 h, which was 1.8 times that of the initial value. Under the stress of 100 μg·L^−1^ and 1000 μg·L^−1^ FF, the EPS content reached the maximum at 192 h, which was 1.1 and 1.2 times that of the initial content, respectively. The EPS content rapidly increased under the stress of 10 μg·L^−1^ and 100 μg·L^−1^ OFL, and reached the highest value at 24 h, which was 1.6 and 1.9 times that of the initial content, respectively. The EPS content increased slowly under the stress of 1000 μg·L^−1^ OFL and reached a peak at 192 h, which was 1.9 times that of the initial content. The EPS content under the higher concentration of antibiotic stress increased slower than those under lower concentrations of antibiotic stress. The results showed that the biofilm EPS content was increased to cope with the stress of FF and OFL. It was reported that the synthesis and secretion of EPS could be promoted when *Microcystis aeruginosa* was exposed to the antibiotic norfloxacin [24]. It was necessary to further elucidate the role of EPS components under the antibiotic stress.

### 3.2. The Interactions of EPS with Antibiotics and the Role of EPS under the Antibiotic Stress

To investigate the role of biofilm EPS under FF and OFL stress, the interaction between the EPS and the two antibiotics was performed by batch sorption experiments. Sorption data were fitted with the Langmuir, Freundlich, and DRDM models. Freundlich models was selected to describe the sorption process due to their higher *r*^2^_adj_ values. The isotherm parameters for FF and OFL sorption by intact and EPS-free biofilms are shown in Table 1. The calculated *K*_d_ of intact and EPS-free biofilms were termed as *K*_d_ and *K*_de_, respectively. The *K*_d_ for FF sorption on intact and EPS-free biofilms were lower than those for OFL.

The sorption of FF and OFL with the selected concentrations and whether it was affected by EPS was compared through the ratios of *K*_de_ to *K*_d_. The *K*_de_/*K*_d_ values for FF and OFL were 3.2 and 6.5, respectively. The results indicated that biofilm EPS could effectively suppress the enrichment of antibiotics on biofilms, and thereby reduce the stress of antibiotics to biofilms. Our previous study also found that EPS inhibited the partitioning and sorption of antibiotic OFL by the biofilms [38]. Both the present and previous studies indicated that EPS acted as a trap for the antibiotics and limited the interaction and adverse effects on biofilms. Moreover, the *K*_de_/*K*_d_ values for the OFL were higher than FF. It indicated that EPS have a stronger inhibitory on OFL binding to biofilms than FF.

**Figure 4:**
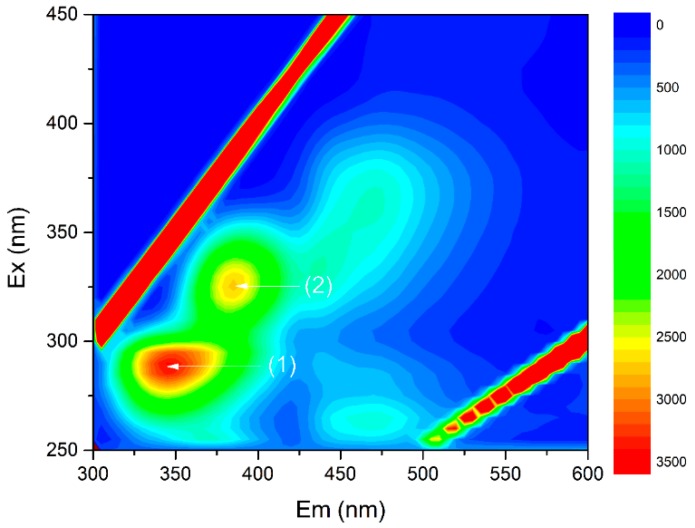
Three-dimensional excitation-emission matrix (3D-EEM) fluorescence spectra of extracellular polymeric substances. Protein-like matters (**1**) and humic-like matters (**2**).

3D-EEM fluorescence spectra and IR spectroscopy were used to analyze the detailed interactions of EPS with FF and OFL. The 3D-EEM fluorescence spectra of the EPS is shown in Figure 4. Two predominant components, including protein-like substances at 285/340 nm and humic-like substances at 320/380 nm, were identified from the spectra. The *K*_q_ values for the protein-like and humic-like substances from the EPS binding with the FF and OFL were summarized in Table 2. Considering that the values were higher than the maximum diffusion collision quenching rate constant of the quencher to the biological macromolecules, i.e., 2.00 × 10^11^ L·mol^−1^·s^−1^, the quenching of EPS fluorescence intensity caused by FF and OFL was mainly attributed to the formation of antibiotic-EPS complexes [44]. The *K*_q_ values of the protein-like substances were much larger than the quenching constant of the humic-like substances component under the FF and OFL effects. It indicated that the protein-like substances dominated the interaction between the EPS and antibiotics. In addition, It was found that the *K*_q_ values from the EPS binding with the OFL were stronger than those with FF, from which it could be deduced that EPS resistance to the OFL stress was stronger than that to FF.

**Figure 5:**
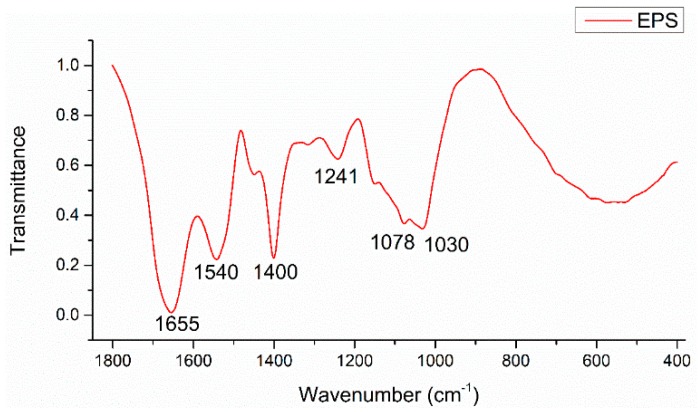
Infrared spectroscopy of the freeze-dried extracellular polymeric substances.

Figure 5 shows the IR spectra of EPS. The band assignments for the IR spectral features of EPS are shown in Table 3. The peaks at 1078 cm^−1^ and 1030 cm^−1^ were assigned to the C–O–C ring vibrations and C–OH stretches derived from polysaccharides. The peak at 1400 cm^−1^ was assigned to the symmetric stretching vibration of carboxyl groups. Moreover, the peaks at 1655 cm^−1^, 1540 cm^−1^, and 1241 cm^−1^ were assigned to the amide І, II, and III vibrations, respectively, stemming from the protein components. The detected C=O functional groups in proteins could combine with antibiotics as reported by Wang, which could prevent the antibiotic invading into biofilm cells [45]. The results showed that proteins in EPS could play an important role in the protection of biofilm cells under the stress of FF and OFL.

## 4. Conclusions

The stress of antibiotic FF and OFL with low and high concentration levels on changes in the chlorophyll, antioxidant enzymes, and EPS of freshwater biofilms was investigated. Results showed that chlorophyll a content in biofilms firstly decreased to 46–71%, and then recovered to original content under the stress of FF and OFL. Meanwhile, The EPS content increased between 1.1–1.9 times their initial values, and the activities of SOD and CAT increased between 1.3–6.7 times their initial values under the stress of FF and OFL. It indicated that biofilms reduced the antibiotic stress through enhancing the EPS content and the activities of SOD and CAT. The distribution coefficients of FF and OFL binding in EPS-free biofilms were 3.2 and 6.5 times higher than those in intact biofilms, respectively. It indicated that EPS prevented the antibiotic invading into biofilm cells. Protein-like substances dominated the interaction between EPS and antibiotics. In summary, biofilms can resist the antibiotic stress through the response of their EPS and enzymes, but EPS play a more critical role in the process.

## Figures and Tables

**Table 1:** Isotherm parameters for antibiotic florfenicol (FF) and ofloxacin (OFL) sorption by intact and extracellular polymeric substances (EPS)-free biofilms.

	Freundlich Model			Distribution Coefficient
Samples	*Q*_e_ = *K*_F_*C*_e_*^n^*			*K*_d_ = *Q*_e_/*C*_e_
	*K*_F_/(mg·g^−1^)·(mg·L^−1^)^−n^	1·n^−1^	*r* ^2^_adj_	*K*_d_/L·g^−1^
FF biofilms	0.48 ± 0.03	1.07 ± 0.15	0.946	0.44 ± 0.07
FF–EPS-free biofilms	1.44 ± 0.26	1.01 ± 0.21	0.890	3.15 ± 0.24
OFL biofilms	0.81 ± 0.03	0.70 ± 0.05	0.982	1.06 ± 0.17
OFL–EPS-free biofilms	10.92 ± 0.81	1.11 ± 0.03	0.997	8.20 ± 0.56

**Table 2:** Stern–Volmer equation fitting results of protein-like and humic-like matters in extracellular polymeric substances.

Components	Forfenicol		Ofloxacin	
	*K*_q_/L·mol^−1^·s^−1^	*r* ^2^	*K*_q_/L·mol^−1^·s^−1^	*r* ^2^
Protein-like matter	1.17 × 10^12^	0.924	4.11 × 10^12^	0.958
Humic-like matter	3.83 × 10^11^	0.980	5.76 × 10^11^	0.985

**Table 3:** Band assignments for Fourier transform infrared spectrometer spectral features (cm^−1^) of extracellular polymeric substances.

Band Position/cm^−1^	Band Assignments
1655	*ν*_s_C=O stretch (amide I) associated with proteins; NH_2_ scissors of primary amines
1540	*δ*N–H and *ν*_s_C–N stretches (amide II) associated with proteins
1400	*ν*_s_COO^−^ stretches associated with amino acids
1241	*ν*_s_C-N stretch associated with secondary amides of proteins (amide III)
1078, 1030	*ν*C-O-C ring vibrations and *ν*C-OH stretches derived from polysaccharides

## Supplementary Materials

The following are available online at https://www.mdpi.com/1660-4601/16/5/715/s1, Information S1: Spectral measurements of extracellular polymeric substances, Table S1: Basic information on florfenicol and ofloxacin, Table S2. Information on the Nanhu Lake and some physicochemical properties of the lake water.
