# Supplementary material for: Response of Freshwater Biofilms to Antibiotic Florfenicol and Ofloxacin Stress: Role of Extracellular Polymeric Substances
Source: Int J Environ Res Public Health. 2019 Feb 27;16(5):715. doi: 10.3390/ijerph16050715 (PMC6427337; doi:10.3390/ijerph16050715)
Supplement: Supplementary file 1 [file ijerph-16-00715-s001.pdf]

Supplementary Material

Response of Freshwater Biofilms to Antibiotic Florfenicol and Ofloxacin Stress: Role of Extracellular Polymeric Substances

Chaoqian Wang, Deming Dong, Liwen Zhang, Ziwei Song, Xiuyi Hua and Zhiyong Guo *

Key Laboratory of Water Resources and Environment, College of New Energy and Environment, Jilin University, Changchun 130012, China; wangcq16@mails.jlu.edu.cn (C.W.); dmdong@jlu.edu.cn (D.D.); zhangliwen@jlu.edu.cn (L.Z); songzw18@mails.jlu.edu.cn (Z.S.); huaxy@jlu.edu.cn (X.H.)

* Correspondence: zyguo@jlu.edu.cn; Tel.: +86-0431-85168429

Information S1 Spectral measurements of extracellular polymeric substances

A part of the above supernatants purified with an 8000-Da semipermeable membrane to remove residues and salt was lyophilized to obtain EPS samples for spectral analysis. The freeze-dried EPS samples were dissolved in Milli Q water, and a series of solutions containing 100 mg·L^{-1} of EPS and 0 to 2.5 mg·L^{-1} of antibiotics were prepared in 40-mL vials. The vials were kept in dark and shaken in a water-bath shaker at 150 rpm for 24 h at 25 ºC. After that, 5 mL of mixture was collected and passed through a 0.45-μm filter for three-dimensional excitation-emission matrix (3D-EEM) fluorescence spectra analysis. The 3D-EEM fluorescence spectra (F-2700, Hitachi, Japan) was measured with subsequent scanning emission wavelength of 300 to 600 nm at 0.5-nm increments by excitation wavelengths of 250 to 450 nm at 10-nm increments. The slits for both excitation and emission were 5 nm, and the scan speed was 1200 nm·min^{-1}.

The freeze-dried EPS samples and KBr were mixed at a ratio of 1:100 and homogenized in an agate grinder. The mixture was compressed into tablets. The interactions between the EPS and the two antibiotics were measured with an infrared spectrometer (IR, IRAffinity-1S, Shimadzu, Japan), and the spectra in the range of 4000 to 400 cm^{-1} were recorded with 20 replications at a resolution of 2 cm^{-1}.

Table S1. Basic information on florfenicol and ofloxacin.

Antibiotic	Molecular structure	CAS Number	Molecular weight	Dissociation Constant	Partition Coefficient	Water solubility
Florfenicol	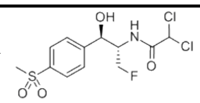	73231-34-2	358.21	9.03	-0.04	1.32
Ofloxacin	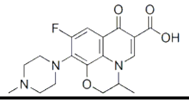	82419-36-1	361.37	6.10/8.28	-0.39	2.55

Table S2. Information on the Nanhu Lake and some physicochemical properties of the lake water.

Latitude (North)	Longitude (East)	Depth Max. (m)	DOC [a] (mg·L^{-1})	TN [b] (mg·L^{-1})	TP [c] (mg·L^{-1})	pH
43°51′	125°18′	6.5	8.61	1.16	0.15	7.4-7.6

[a] DOC is the dissolved organic carbon.

[b] TN is the total nitrogen.

[c] TP is the total phosphorus.
